# An Interactive Digital Dashboard for Patient Monitoring and Management in a Continuity of Care Centre: Development and Preliminary Usability Evaluation Study

**DOI:** 10.2196/81480

**Published:** 2026-06-12

**Authors:** Chiara Dachena, Roberto Gatta, Lorenzo Franceschini, Mario Antonio Del Vicario, Maria Letizia Serra, Nicola Acampora, Antonio Marchetti, Giovanni Arcuri, Stefano Patarnello, Carlotta Masciocchi, Graziano Onder, Francesco Landi, Christian Barillaro

**Affiliations:** 1 UOS Data Science - Gemelli Generator RWD Fondazione Policlinico Universitario Agostino Gemelli IRCCS Rome Italy; 2 Department of Clinical and Experimental Sciences University of Brescia Brescia Italy; 3 Faculty of Economics Catholic University of the Sacred Heart Rome Italy; 4 Department of Geriatric, Orthopedic, and Rheumatological Sciences Fondazione Policlinico Universitario Agostino Gemelli IRCCS Rome Italy; 5 Fondazione Policlinico Universitario Agostino Gemelli IRCCS Rome Italy

**Keywords:** clinical dashboard, continuity of care center, health information technology, Questionnaire for User Interaction Satisfaction, QUIS, SAI, Situation Awareness Index, SUS, System Usability Scale

## Abstract

**Background:**

Clinical dashboards are becoming important tools for managing and monitoring hospitalized patients across different wards. Moreover, careful attention to design, usability, and user interaction is essential for developing effective support tools for clinicians.

**Objective:**

This study aimed to describe the development, implementation, and preliminary evaluation of an interactive dashboard for patient monitoring and management in the Continuity of Care Centre (CCC).

**Methods:**

We developed a dashboard according to clinicians’ requests and the daily workflow of case managers. First, a CCC Data Mart was created to collect all patients’ information automatically extracted from the hospital’s data warehouse. However, case managers had the possibility to enter additional patient information in the dashboard using a dedicated form. Moreover, CCC physicians, nurses, and administrative staff were surveyed using 2 validated questionnaires, the System Usability Scale and the Questionnaire for User Interaction Satisfaction. The Situation Awareness Index was proposed to evaluate user awareness and task efficiency.

**Results:**

The first version of the CCC dashboard presented 4 panels with different types of information, both on the individual patient and on metrics related to the overall patient population. The first panel focused on patients’ data, such as demographic factors, admission, transfer, discharge wards (and their dates), etc. The importance of this panel was the possibility of viewing information collected from different sources within a single interface. The other 3 panels displayed different key performance indicators for the overall patient population and presented data both in the form of tables in the second panel and graphs in the third and fourth panels. After 3 months of daily use, a total of 15 participants, 10 nurses, 2 administrative staff members, and 3 physicians, were recruited for the dashboard evaluation. The average System Usability Scale score of the dashboard was 61.5 (SD 15.7) points, which indicates “OK to good” usability, and the median score obtained with the Questionnaire for User Interaction Satisfaction was 5.77 (IQR 4-7), with the highest results in usability (mean 6.33, SD 0) and learning (mean 6.01, SD 0.39). The overall Situation Awareness Index score was 4 points, with the highest score in “familiarity of dashboard” (mean 4.73, SD 1.66 points) and “arousal support” (mean 4.6, SD 1.8 points).

**Conclusions:**

We developed an interactive dashboard for patient monitoring and management, with positive evaluations from users across different questionnaires.

## Introduction

### Prior Work

Clinical dashboards represent useful tools to monitor and manage patients during hospitalization. In the past years, several dashboards were developed for different diseases and departments [1-3] to improve the management of patients and their quality of care. Dashboards allow the integration of several patient information from different data sources, such as hospitalization data, laboratory data, radio diagnostic data, electronic health records (EHRs), etc, to obtain a compact visual representation of data. Relevant information grouped on a dashboard can be used to help clinicians in the decision-making process.

The growing complexity of patient care in continuity of care centers demands advanced digital solutions to enhance monitoring and coordination. In this regard, increasingly, health care organizations are introducing dashboards as an efficient way of measuring and improving the quality of care provided by their organizations.

To address these challenges, dashboards are now starting to be considered essential tools in modern medical practice, delivering real-time integration, easy visualization, and workflow efficiency in clinical practice. By facilitating multidisciplinary collaboration, improving workflow efficiency, and enhancing patient management, dashboards foster greater adherence to clinical guidelines, improved patient safety, and quality care outcomes. Strong evidence suggests that implementing clinical and quality dashboards that offer clinicians immediate access to information can enhance adherence to quality guidelines [4,5].

The effectiveness and quality of these tools were assessed in a systematic review by Dowding et al [4] that examined the use of clinical and quality dashboards in health care settings. The study highlighted that dashboards provide timely and relevant information, aiding decision-making and improving patient care. Another example was provided by Siette et al [6], who conducted a comprehensive systematic review analyzing the effectiveness of dashboards in aged care environments, highlighting that those features, such as bar charts, interactive displays, and real-time reporting, significantly contribute to clinician satisfaction and usability. Bucalon et al [7] proposed a scoping review aimed at summarizing the literature on dashboards based on administrative, medical, and surgical data for clinicians to support reflective practice.

The integration of clinical dashboards and clinical decision support systems has been shown to significantly enhance workflow efficiency, communication, and patient outcomes in complex medical decision-making environments. Clarke and Wilson [8] highlighted that real-time dashboards improve the efficiency within a specific clinical setting, enabling them to process over 150 patient data points efficiently, reducing cognitive overload and minimizing the risk of errors.

Together, these studies reinforce the critical role of user-centered design, real-time data visualization, and intuitive interface features in improving the adoption and effectiveness of clinical dashboards in aged care. By aligning dashboard functionalities with the specific needs of aged care providers and caregivers, these tools can enhance workflow efficiency, patient monitoring, and overall care outcomes, ultimately contributing to a more sustainable and data-driven aged care system. With ongoing advancements in data analytics, artificial intelligence, and user-centered design, their role in shaping the future of digital health will only continue to expand, also thanks to further research in this field.

### Goal of This Study

This study is a pilot implementation and a preliminary evaluation of a clinical dashboard in a large Italian hospital for monitoring, management, and clinical support. In our work, we developed a personalized clinical dashboard for use in the Fondazione Policlinico Universitario A Gemelli IRCCS’s Continuity of Care Centre (CCC), with a view to enhancing patient tracking, care coordination, supporting decision-making in a clinical environment, and integrating alerts to promptly identify critical clinical situations. This unit was created in 2016 with 1558 beds and 41 operating rooms [9], and it is composed of multidisciplinary professionals, medical doctors, and nurses skilled in case management. The aim of this group was to guarantee safer and faster hospital discharge for frail patients, reduce hospital length of stay, counteract unplanned readmission to the hospital, and improve the coordination of postacute health care services. The CCC is activated by hospital wards through an order or by default if the Blaylock Risk Assessment Screening Score (BRASS) is ≥20. Given the importance of the CCC in the hospital and the high number of patients treated every day, it was necessary to provide a tool that could facilitate the organization and management of patients. In fact, before the dashboard was developed and started to be used, patients were managed using a Microsoft Excel file. This workflow was complex and problematic.

The dashboard design and development were conducted in close collaboration with clinicians because they had a specific workflow, and the proposed tool is an improvement over what was already in use. For this reason, the presented system was not informed by previously cited works, but an in-house approach, described better in the next paragraphs, was followed.

In a real-life environment, over a period of more than 1 year in which the system was daily used, our dashboard exhibited a significant impact in terms of workflow efficiency, tracking, and interprofessional communications. To assess its usability, effectiveness, and clinical impact, we used 2 validated assessment tools: the System Usability Scale (SUS) and the Questionnaire for User Interaction Satisfaction (QUIS). Moreover, the Situation Awareness Index (SAI) is proposed. These provided a comprehensive assessment of user experience, with measures such as ease of navigation, responsiveness, cognitive workload, and perceived efficiency. The findings provided valuable feedback on system performance, guiding ongoing development and ensuring the dashboard remains fit for purpose to address clinician requirements and patient care objectives.

## Methods

This study adhered to the iCHECK-DH (Guidelines and Checklist for the Reporting on Digital Health Implementations) checklist [10].

### CCC Data Mart

This study aimed to develop a system that would facilitate the management and monitoring of CCC patients. The development and implementation of the dashboard were performed by the Gemelli Generator [11] at the Fondazione Policlinico Universitario A Gemelli IRCCS, Rome, Italy, in close partnership with the CCC. The Gemelli Generator is responsible for extracting, standardizing, and integrating data collected from different views provided by the hospital’s data warehouse (DWH). The aim is to build standardized and structured archives, called Data Marts, for specific research areas to conduct projects and to develop applications. The Data Mart was based on an ontology defined with the clinicians and includes all the useful variables considered in clinical practice. After defining the variables, specific extraction, transformation, and loading procedures were developed for structured and unstructured data to produce tables that include all relevant information about the patients. Patients signed an informed consent to collect and use their data retrospectively for scientific purposes.

**Figure 1:**
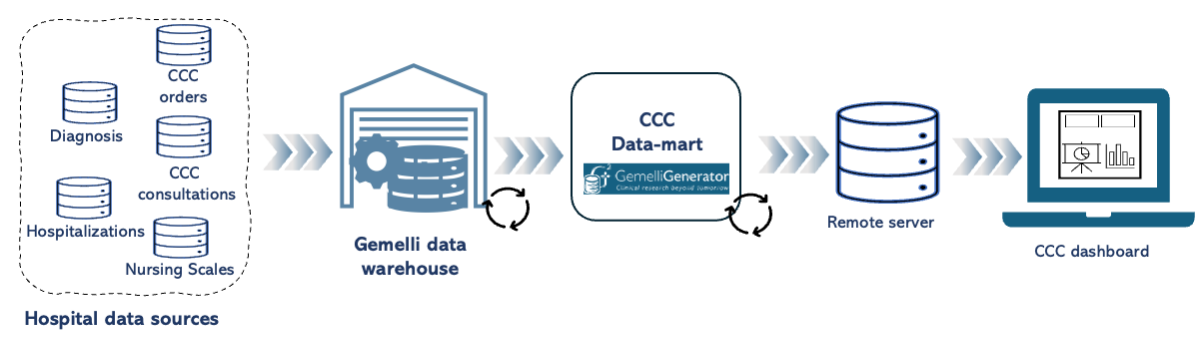
Gemelli Generator workflow to create and automatically update the Continuity of Care Centre (CCC) Data Mart, from hospital data sources to the dashboard.

The CCC Data Mart consists of all patients with at least 1 CCC consultation or an order during hospitalization. All data related to the hospitalization were collected, such as demographic factors, admission, transfer, and discharge wards (and their dates), admission diagnosis, BRASS scales, and CCC consultation texts. The Data Mart was updated twice daily (at 9 AM and 2 PM), and the procedures linked the data directly from the EHR. In this way, it was possible to visualize the data near real time, integrating new information about patients who were already hospitalized and adding new patients who were admitted during the day. The workflow to create and automatically update the CCC Data Mart, starting from the hospital data sources to the dashboard, is presented in Figure 1.

The final product and intellectual property remained under the ownership of Gemelli Generator after implementation.

### Dashboard Design

To design the initial dashboard prototype, we started by analyzing the existing daily workflow of case managers. Every day, during a morning briefing, the CCC nurses planned their daily activities and identified the candidate patients who would be visited during the day. During the clinical interview, they collected all patients’ information on a nursing diary, such as clinical and personal data, and subsequently recorded the information in the EHR.

**Figure 2:**
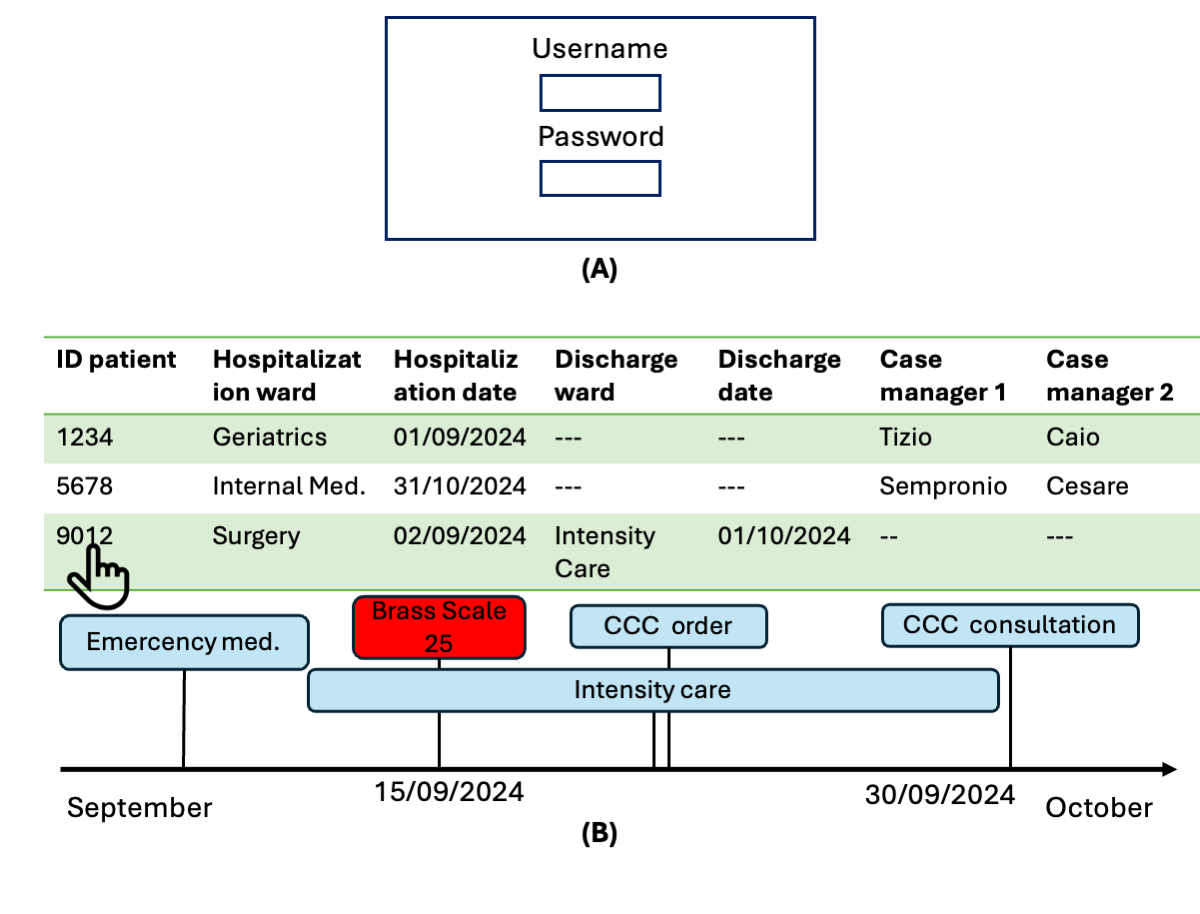
Mockup of the proposed dashboard prototype. (A) A log-in page that allows users to insert their credentials. (B) The first page shows a table in which each row represents a patient, and each column represents patient-related information.

**Figure 3:**
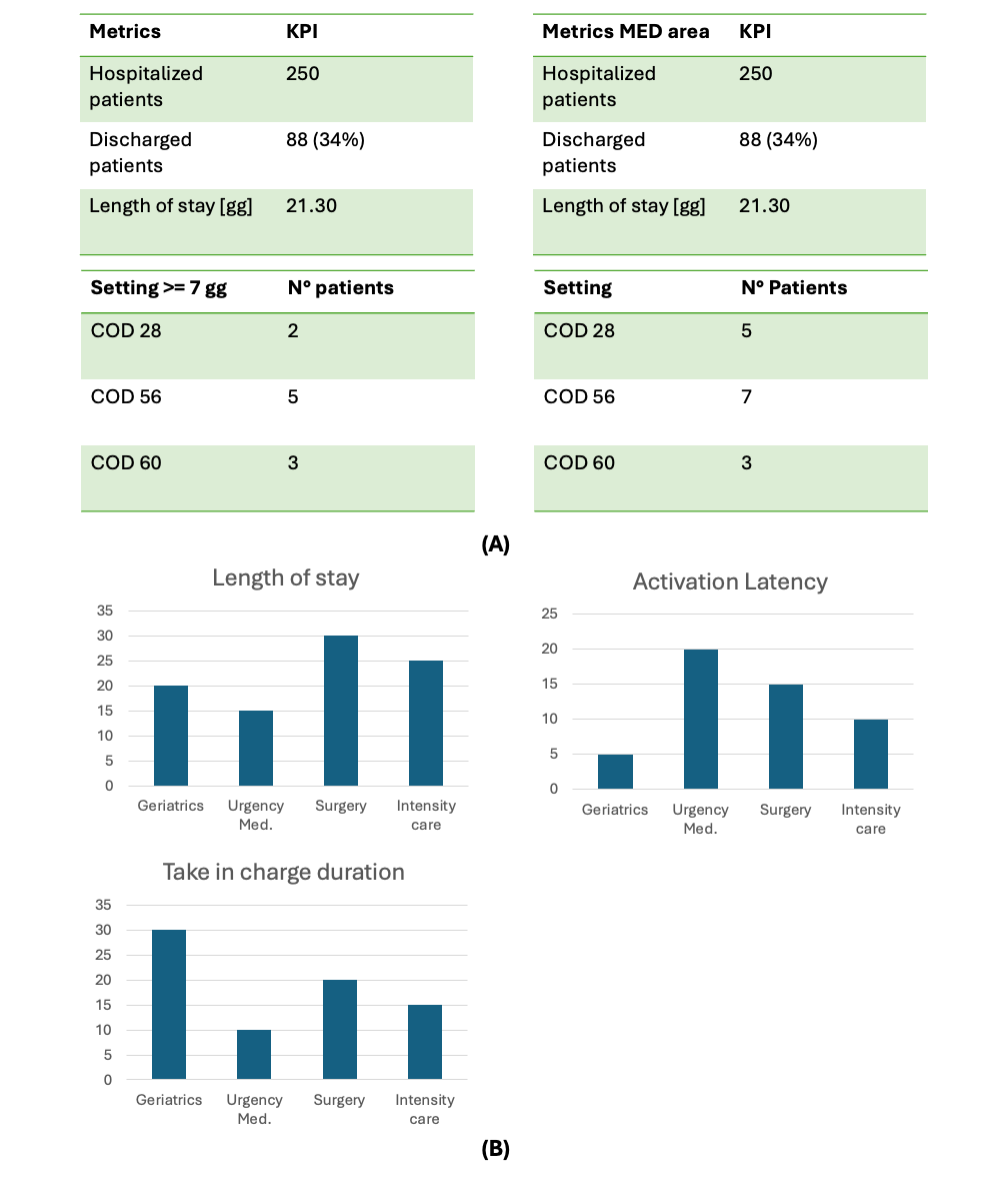
Mockup of the proposed dashboard prototype. (A) Second tab with key performance indicators (KPIs) tables. The proposed KPIs described the number of hospitalized patients, discharged patients, and the median length of stay. (B) Third tab with proposed graphs, such as the length of stay of each ward, the activation latency, and the take in charge duration.

To manage and monitor the activity of the CCC, a shared Microsoft Excel file, accessible to all case managers, was used to collect all patients in charge and to immediately visualize the patients’ situation. In this file, they integrated all patient information, such as patients’ ID, admission data, etc. This process required a lot of time for the clinicians, who had to be careful to correctly enter the information, especially the numeric data. This workflow increased the risk of data-entry errors because of the large number of patients managed every day. Furthermore, it is important to emphasize the difficulty of tracking patients’ movements during transfers between different departments, considering the size of the hospital; in fact, to know the ward in which the patient was hospitalized at a specific time, it was necessary to search for it in the hospital system. To propose a dashboard that would be useful and easy to manage by the nurses, we jointly analyzed the Excel file to understand what each column represented and if the data should be automatically extracted from the DWH. First, we proposed a simple table, which replicated the data and the functions of the Excel file. We identified which data could be extracted automatically from the DWH and which had to be inserted manually by the nurses. As expected, most data are derived from the DWH; this was an important step because it allowed nurses to reduce the time spent compiling the file and to focus more on the patients. The second step was to meet with the nurses to identify the crucial aspects in their daily workflow and what should improve the patients’ evaluation. We proposed the possibility of having the patients’ timeline with all important information, such as the transited wards, the BRASS scale trends, and the text of the CCC consultations. This improvement in the dashboard allowed the clinicians to have useful information before visiting the patients and, within the same view, without moving to the EHR or from one archive to another in the medical record to retrieve information. Finally, at the request of the CCC managers, we developed a panel with several important key performance indicators (KPIs), such as the total patients in charge, the discharged patients, etc, and a panel with graphics that showed different important statistics. In Figure 2, the proposed dashboard mockup is shown, with a log-in page and a simple view of the first tab with the patients’ table, with the requested data and timeline. In Figure 3, the second tab presents important KPIs as a table, and the third tab presents different graphs.

### Dashboard Implementation

The dashboard was developed and implemented specifically for this study. We implemented the dashboard using the Shiny R library [12] on an Apache web server [13] running on a secure and private virtual server. The server could be accessed over the hospital’s private network after user authentication, and different permissions were created depending on the user. We proposed 3 different user types: clinicians, technicians, and administrative staff. Technicians could not visualize the patient’s personal data, such as name, CCC consultation texts, etc, and could not modify the data. Clinicians and administrative staff could visualize all types of data, and differed from each other because the latter could modify user permissions. These different user types were managed through Lightweight Directory Access Protocol authentication [14] with hospital credentials. This protocol accommodates the need for a high level of security and centralized user management. The clinical dashboard was updated twice daily, in the morning to include the information of the day before, and in the afternoon to include the information collected during the morning round; this guaranteed effective and efficient use of the dashboard during the daily routine. In fact, beyond the layout, usability was the most important feature to consider during the implementation phase due to the dashboard’s integration in the clinical practice.

**Figure 4:**
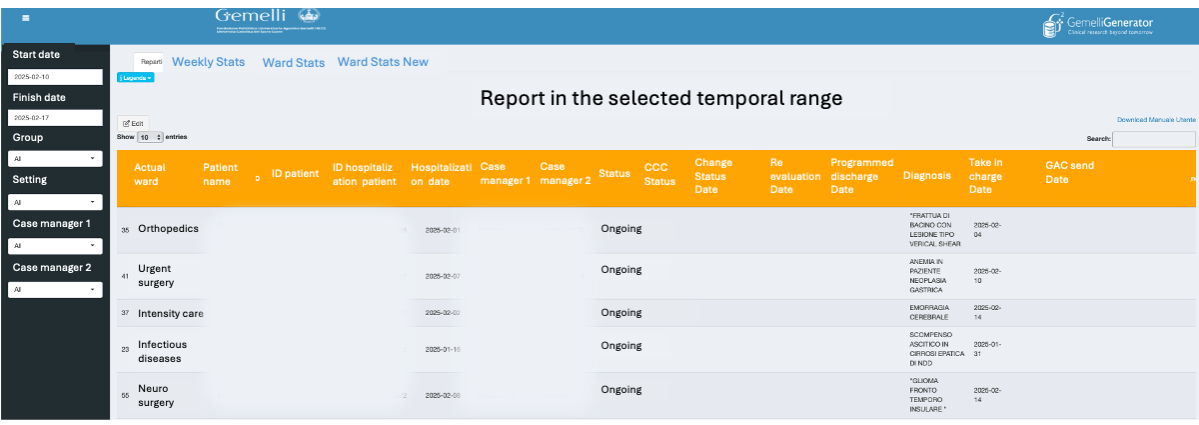
First panel of the developed clinical dashboard. Each row represents a patient with several information items, such as patient ID, actual ward, status, etc.

The dashboard includes 4 panels, each containing relevant information for the case managers. In Figure 4, the principal panel is shown. On the left side, a sidebar with filtering options allows clinicians to select different subgroups of patients. The first filter allows users to select the time range in which to visualize the patients in charge, with start and finish dates. The other filters allow users to filter patients with specific features, such as patients hospitalized in medical wards, patients with a specific discharge setting, and the first and second case managers responsible for the patients. These filters modify the list of patients shown in the center of this panel.

**Figure 5:**
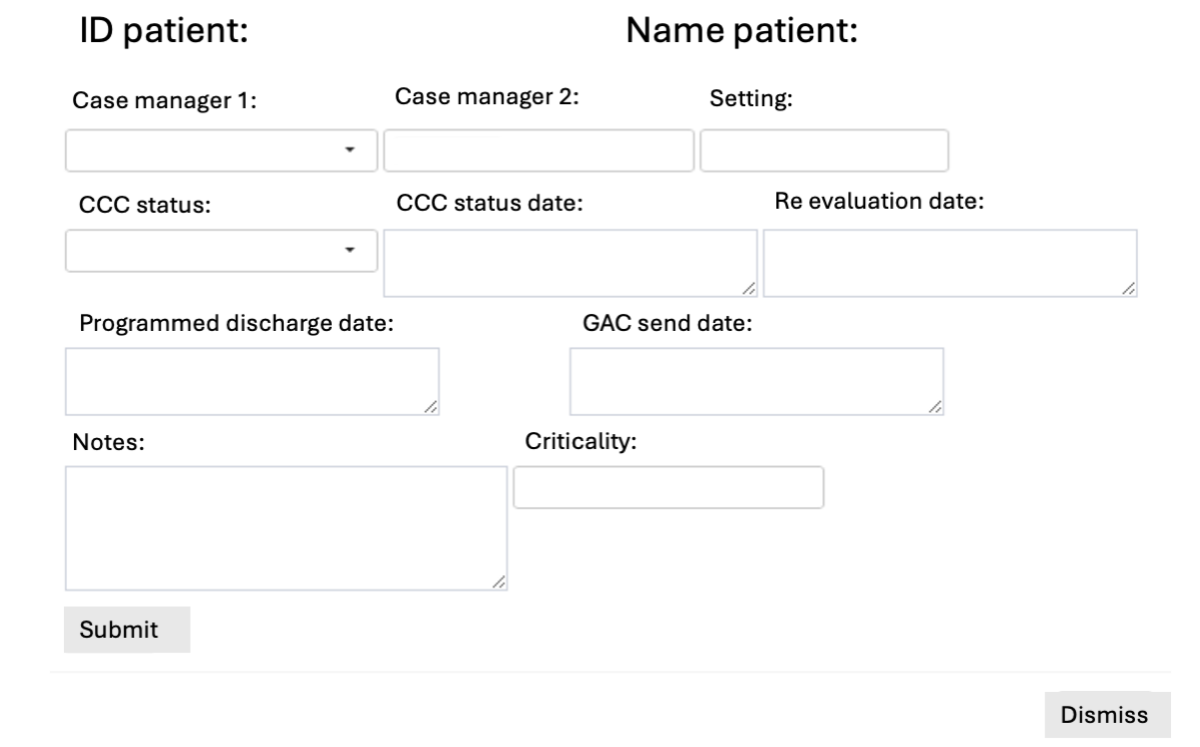
Pop up with an insert form. Different information can be added via a dropdown menu or free text.

Each row of the list represents one patient and their features, such as the identification data, the admission date, and the ward, etc. For each patient, 28 items were collected, 23 (82%) of which were automatically extracted. For the remaining 5 (18%) fields, which represent information not presented in the information system, we implemented a pop-up form to allow the clinicians to insert the other patients’ information, as shown in Figure 5. In this form, the case managers could insert several additional information items by a dropdown menu, such as the discharge setting and a possible change of patient status, or by a free-text field, such as different dates, patient criticality, and other notes. Moreover, they could change the first and second case managers. This extra information, inserted by the case manager, was saved in an SQL database on the virtual server previously mentioned. Each considered variable is explained in a structured overview in Table S4 in Multimedia Appendix 1. For each variable, the format and input type are reported. The table implements some visual alerts: yellow highlights patients with a palliative consultation, whereas green and gray highlight discharged and deceased patients, respectively.

**Figure 6:**
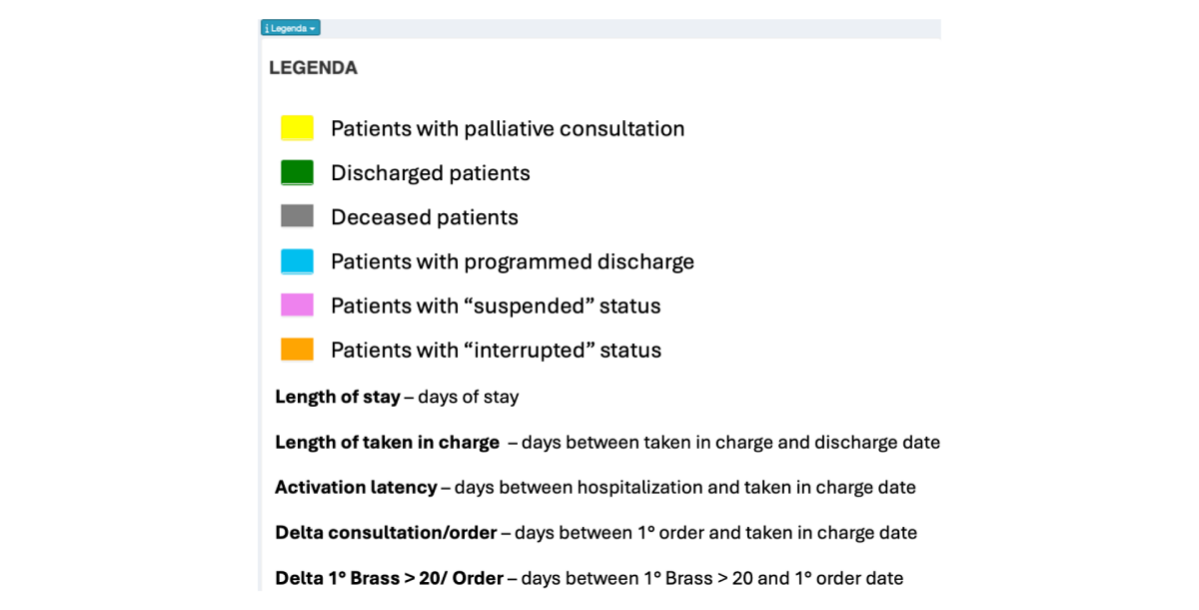
Legend with all possible visual alerts. Each color represents a different patient’s critical issue.

**Figure 7:**
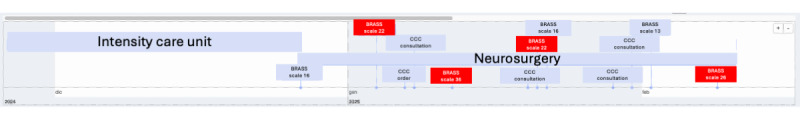
Patient’s timeline. Each CCC consultation, Blaylock Risk Assessment Screening Score scale, CCC order, and transited ward is presented in a compact view.

All possible alerts are explained in Figure 6. These visual alerts allow clinicians to more easily identify patients who need more attention during clinical practice, without reading each patient’s clinical diary. Finally, by clicking on a patient row, the hospitalization timeline is shown with all important information and with the possibility to visualize the texts of CCC consultations (Figure 7). In this panel, it is also possible to download the user manual, which describes the dashboard’s functions and the information presented in each panel. The proposed user manual described step by step how to navigate the dashboard and how to use it. In fact, starting from the log-in page, each dashboard page was well described, for example, the data filters, the insert form, etc. The manual was always available to the clinicians to assist them if they encountered difficulties during use.

**Figure 8:**
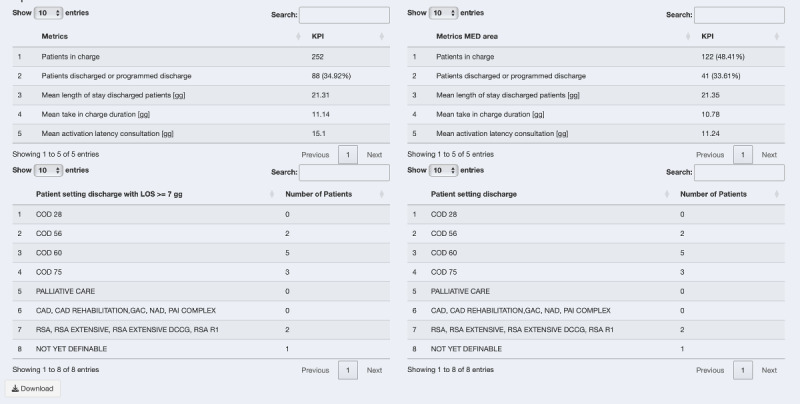
Second panel of the clinical dashboard. Tables at the top represent general patients’ metrics; tables at the bottom represent the number of patients for some of the settings of discharge.

The second panel shows 4 tables with different KPIs, as shown in Figure 8; the top tables report metrics on patients in charge and discharged patients, including length of stay, time to assignment, and activation latency. The right table isolates data for patients admitted to medical wards. The bottom tables summarize discharge settings for in-charge patients; the left table focuses on those with an average stay of approximately 7 days. These tables are important for monitoring and managing CCC patients, to have a complete overview of the patients in charge and to understand the most likely discharge settings. Moreover, it is possible to identify the most complex patients that needed more attention during hospitalization.

In this panel, there is also a button to download a PDF report file that includes the principal information about the patients. This button is activated just for the CCC managers to monitor the activity.

**Figure 9:**
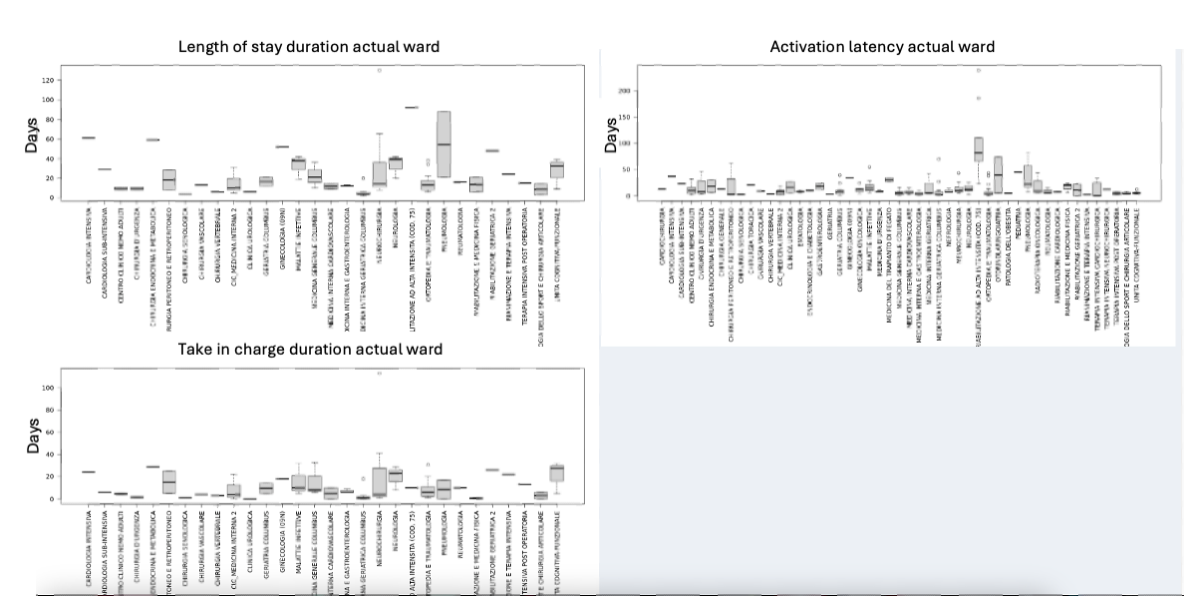
Third panel with 3 different graphs. Each graph represents a metric for different actual wards.

**Figure 10:**
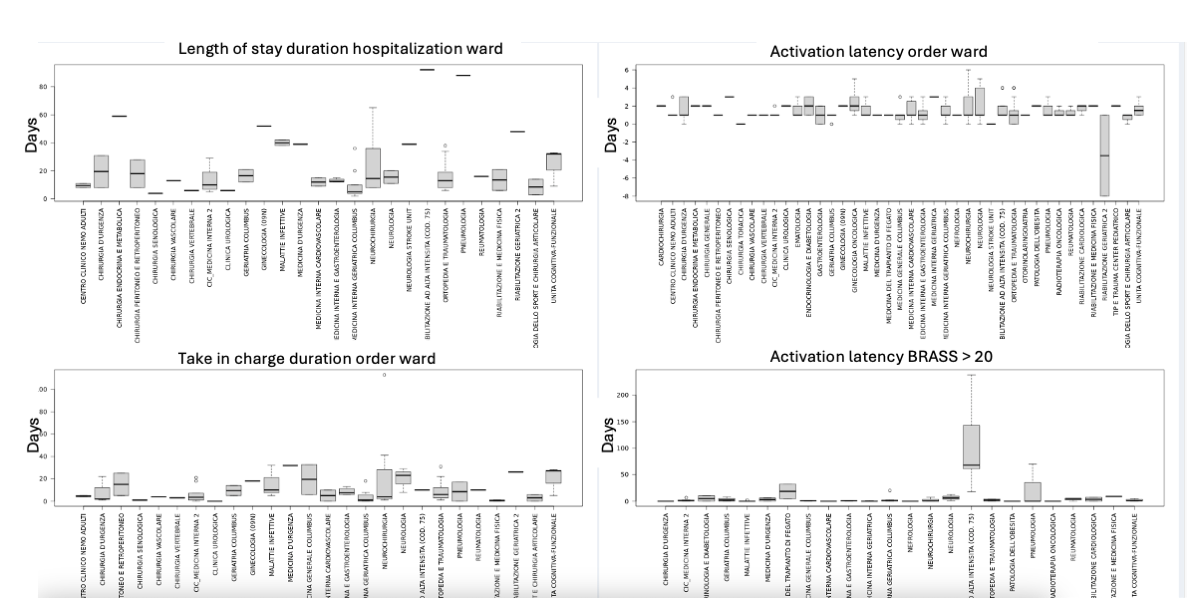
Fourth panel with 4 different graphs. Each graph represents a metric for different wards, such as the hospitalization ward and the order ward.

The third and fourth panels include different graphics presenting ward-related statistics. Specifically, in the third panel, there are 3 charts that show the length of stay, the activation latency, and the duration of taking in charge for current ward. The charts are bar plots that explain the patient distribution for each ward, highlighting possible outcomes (Figure 9). The last panel was developed by the Data Science Unit to highlight another important perspective of the patient population. In fact, the first 3 charts present the same information as the previous panel, but in this case, the examined ward is not the current one and differs for each graph. Specifically, the length of stay is calculated for the admission ward, the activation latency, and the length of taking in charge for the ward that sent the order. Another graph is proposed: the activation latency in cases of the BRASS scale scores ≥20. In this case, the analyzed ward is that in which the BRASS scale was evaluated. This last panel, in our opinion, can be useful for the CCC managers to get a more complete view of the patients’ organization (Figure 10).

### Dashboard Evaluation

In order to have efficient dashboards for clinical and managerial decision-making, these tools should include the following:

- All the requested components
- Few or no usability problems
- A good user interaction
- User awareness and task efficiency

To evaluate if the developed dashboard included these requirements, we used different approaches:

- All requested components: the proposed fields and dashboard layout were evaluated with repeated exchange of feedback with the clinicians, who highlighted any missing elements or required modifications in the dashboard.
- Few or no usability problems: the usability of the dashboard was evaluated using a validated 10-item SUS questionnaire [15-18].
- Good user interaction: the interaction satisfaction was evaluated with the validated short form 50-item QUIS [19].
- User awareness and task efficiency: the 2 aspects were evaluated with SAI [20].

Both SUS and QUIS questionnaires were proposed in their validated Italian version.

The SUS is a versatile questionnaire designed to evaluate any type of technology and is generally quick and easy to complete. It consists of 10 statements, each rated on a 5-point scale based on the strength of agreement, with the final scores ranging from 0 to 100. A higher score suggests better usability. As a general guideline, any score above 70 is considered to have “good” usability, while scores below 70 are considered to indicate “poor usability,” and the system requires further evaluation and improvements [16,18,21]. Textbox 1 presents the proposed questions.

**Questionnaire proposed to the clinicians to assess the usability of the dashboard [15].**

- I think I would like to use this system frequently
- I found the system unnecessarily complex.
- I thought the system was easy to use
- I think I would need the support of a technical person to be able to use this system
- I found the various functions in this system were well integrated
- I thought there was too much inconsistency in this system
- I would imagine that most people would learn to use this system very quickly
- I found the system very cumbersome to use
- I felt very confident using the system
- I needed to learn a lot of things before I could get going with this system

The QUIS, developed by a team of researchers at the University of Maryland, specifically measures user satisfaction with various aspects of a technology system. This includes overall satisfaction with the system as well as specific elements such as screen design and system terminology. The questionnaire is adaptable to focus on user interface analysis, and for this study, the shortened version of the QUIS was used. The questionnaire includes the subcomponents most relevant to the dashboard evaluation, which include the following parts: overall user reactions, screen design, terminology and dashboard information, learning, system capabilities, usability, and UI. Clinicians evaluated the dashboard on a scale from 1 to 9 for each subcomponent. Textbox 2 presents the selected questionnaire’s subcomponents.

**Questionnaire proposed to the clinicians to assess the interaction satisfaction [19].**

**Overall reaction to the software**

- Terrible/Wonderful
- Difficult/Easy
- Dull/Stimulating
- Rigid/Flexible

**Screen**

- Characters on the computer screen: hard to read/easy to read
- Highlighting on the screen simplifies task: not at all/very much
- Organization of information on screen: confusing/very clear
- Sequence of screens: confusing/very clear

**Terminology and system information**

- Use of terms throughout system: inconsistent/consistent
- Computer terminology is related to the task you are doing: never/always
- Position of messages on screen: inconsistent/consistent
- Messages on screen which prompt user for input: confusing/clear

**Learning**

- Learning to operate the system: difficult/easy
- Exploring new features by trial and error: difficult/easy
- Remembering names and use of commands: difficult/easy
- Tasks can be performed in a straight-forward manner: never/always
- Supplemental reference materials: confusing/clear

**System capabilities**

- System speed: too slow/fast enough
- System reliability: unreliable/reliable

**Usability and UI**

- Use of colors and sounds: poor/good

The SAI was developed to evaluate the effectiveness of interfaces in maintaining user awareness and task efficiency [20].

This section includes 5 questions. Participants rate each question on a scale, usually from 1 (strongly disagree) to 7 (strongly agree). The score calculation involves using the SAI formula: SAI = Q1+ Q2 + (6 – Q3) + Q4 + Q5/5, where Q1-Q5 are the mean scores for each question, and Q3 is adjusted to account for distractions. Q3 considers the reversal of the negative impact: for questions related to distractions (eg, “The dashboard distracts attention from important tasks”), the responses are adjusted to reflect a positive contribution to the SAI. This is done by subtracting the score from a constant to reverse the negative impact [2,22].

If the overall SAI is above 4, it suggests a relatively strong awareness, aligning with effective performance standards in complex environments. However, any score close to or below 3 might indicate a need for improvement in situational awareness elements.

Considering that the SAI has not been validated, we decided to translate the questions into Italian ourselves to make them more understandable for clinicians, as shown in Textbox 3.

**Questionnaire proposed to the clinicians to assess the task efficiency [20,22].**

**Instability representation**

- The dashboard adequately represents the instability of the CCC?

**Arousal support**

- The dashboard helps me be alert and clearer?

**Division of attention**

- The dashboard distracts attention from important tasks of the CCC?

**Information quantity provided**

- The quantity of information provided by the dashboard is appropriate for performing CCC tasks?

**Familiarity of dashboard**

- I can perform CCC tasks more proficiently using the dashboard?

### Participant Characteristics

A total of 15 participants were recruited for the dashboard evaluation. All worked in the CCC and, at that moment, represented the entire workforce of the unit. They included 10 nurses, 2 administrative staff members, and 3 physicians, predominantly female (n=10, 66.6%).

The dashboard was evaluated by the participants after 3 months of use.

Before officially starting to use the dashboard, practical sessions were organized with all participants to explain how to use it. In these sessions, the developers demonstrated all the dashboard’s features step by step, starting with the log-in, and showing practical examples of use, such as searching for a patient, entering data via the pop-up form, viewing consultation texts, etc. These sessions were conducted interactively, and all participants were invited to actively participate using the dashboard and to ask questions if any steps or usage were unclear. At the end of these sessions, all participants were able to access the dashboard, use the filters, search for patients, and insert information in the form.

### Budget and Sustainability

The design and development of the CCC dashboard required an estimated effort of 40 full-time days. This project was fully developed by the internal facility of Gemelli Generator, and for this reason, it was self-financed. Sustainability is ensured through the constant partnership between Gemelli Generator and CCC, which includes updates to ensure compatibility with evolving operating systems and periodic revisions. Long-term plans will include a second dashboard release, with the proposed modification and different program languages.

### Ethical Considerations

The study conforms with the European General Data Protection Regulation directives and consequent Italian legislation references (European Union Directive 2016/679 and under Italian Laws: Decreto Legislativo 196/2003, Decreto Legislativo 101/2018, and Autorizzazione Generale Garante 9/2016). In line with the General Data Protection Regulation directives and the designation of Policlinico Gemelli Hospital as a National Center for Health Research and Care from the Italian Ministry of Health, observational studies do not require a specific request for patient consent. All technical procedures and security measures have been reviewed and approved in the context of a data protection impact assessment from the hospital data privacy unit and data protection officer. This study received approval from the Ethics Committee of Policlinico Gemelli to conduct the presented research (protocol number 0009272/23). All clinician participants did not receive payment or compensation for completing the questionnaires.

## Results

### Overview

The developed dashboard includes only patients taken in charge by the CCC at Fondazione Policlinico Gemelli in Rome. Considering the time range from October 1, 2021, to December 31, 2024, the CCC took charge of 11,740 patients with 13,753 hospitalizations, performed 12,920 consultations, and received 13,508 orders, with a mean of 70 (SD 15) monitored patients each week. This large number of heterogeneous patients, together with the fragmentation of patient information within the EHR, made it difficult to manage and organize the case manager workflow. For this reason, there was a need to develop an interactive dashboard that could automatically collect most patient information within the same view.

### SUS Evaluation

**Figure 11:**
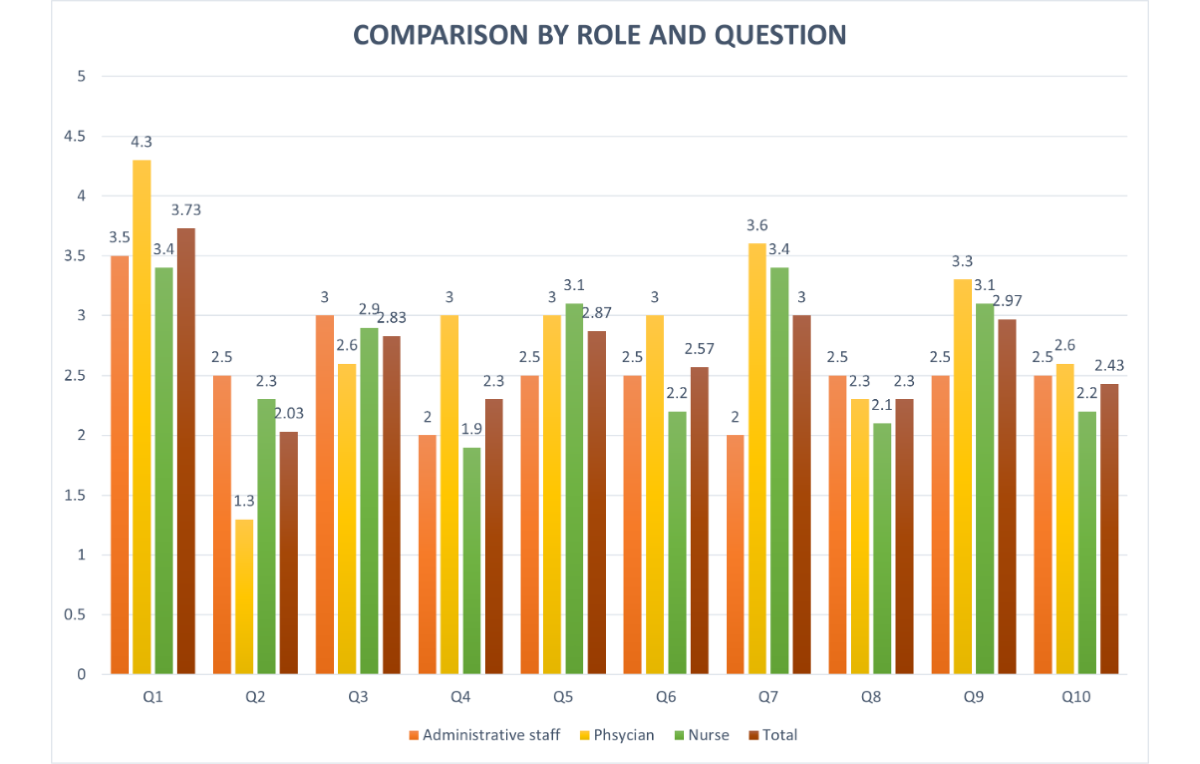
Comparison by patient subgroup and the question of System Usability Scale. For each question, the evaluation for each user group and the total study population is presented.

The mean SUS score of the CCC dashboard was 61.5 (SD 15.7). The SUS score showed a slight difference between the 3 groups; the nurse group had a mean score of 63 (SD 18.2), the administrative staff had a mean score of 53.75 (SD 5.3), and the physician group had a mean score of 61.6 (SD 11.8), as shown in Table 1. The results can be interpreted differently among the 3 principal user groups; for nurses and physicians, the acceptability range of the dashboard was “marginally high,” and the adjective rating was “OK to good,” whereas for administrative staff, the acceptability range was “marginally low” and the adjective rating was “OK” [16]. These findings highlight that nurses and physicians used the dashboard more frequently than administrative staff and considered it a useful tool for monitoring patients in charge. Each user group’s result, grouped by question, is shown in Figure 11. As can be seen, all physician evaluations were higher than those of the other 2 groups.

**Table 1:** System Usability Scale (SUS) scores obtained for different user groups and for the total study population.

SUS item	Nurse, mean (SD)	Physician, mean (SD)	Administrative staff, mean (SD)	Total, mean (SD)
Use frequency	3.4 (1.0)	4.3 (1.1)	3.5 (0.7)	3.6 (1.0)
Complexity	2.3 (0.9)	1.3 (0.5)	2.5 (0.7)	2.1 (0.9)
Ease of use	2.9 (1.2)	2.6 (0.5)	3 (0)	2.8 (1.0)
Technical support needed	1.9 (0.8)	3 (0)	2 (1.4)	2.1 (0.9)
Integration of functions	3.1 (1.2)	3 (1)	2.5 (0.7)	3 (1.1)
Inconsistency	2.2 (1.0)	3 (1)	2.5 (0.7)	2.4 (0.9)
Ease of learning	3.4 (0.9)	3.6 (1.1)	2 (1.4)	3.2 (1.0)
Cumbersomeness	2.1 (0.9)	2.3 (0.5)	2.5 (0.7)	2.2 (0.8)
Confidence using system	3.1 (1.3)	3.3 (0.5)	2.5 (0.7)	3.0 (1.1)
Need to learn before use	2.2 (0.9)	2.6 (0.5)	2.5 (0.7)	2.3 (0.8)
Total	63 (18.2)	61.6 (11.8)	53.75 (5.3)	61.5 (15.7)

### QUIS Evaluation

**Figure 12:**
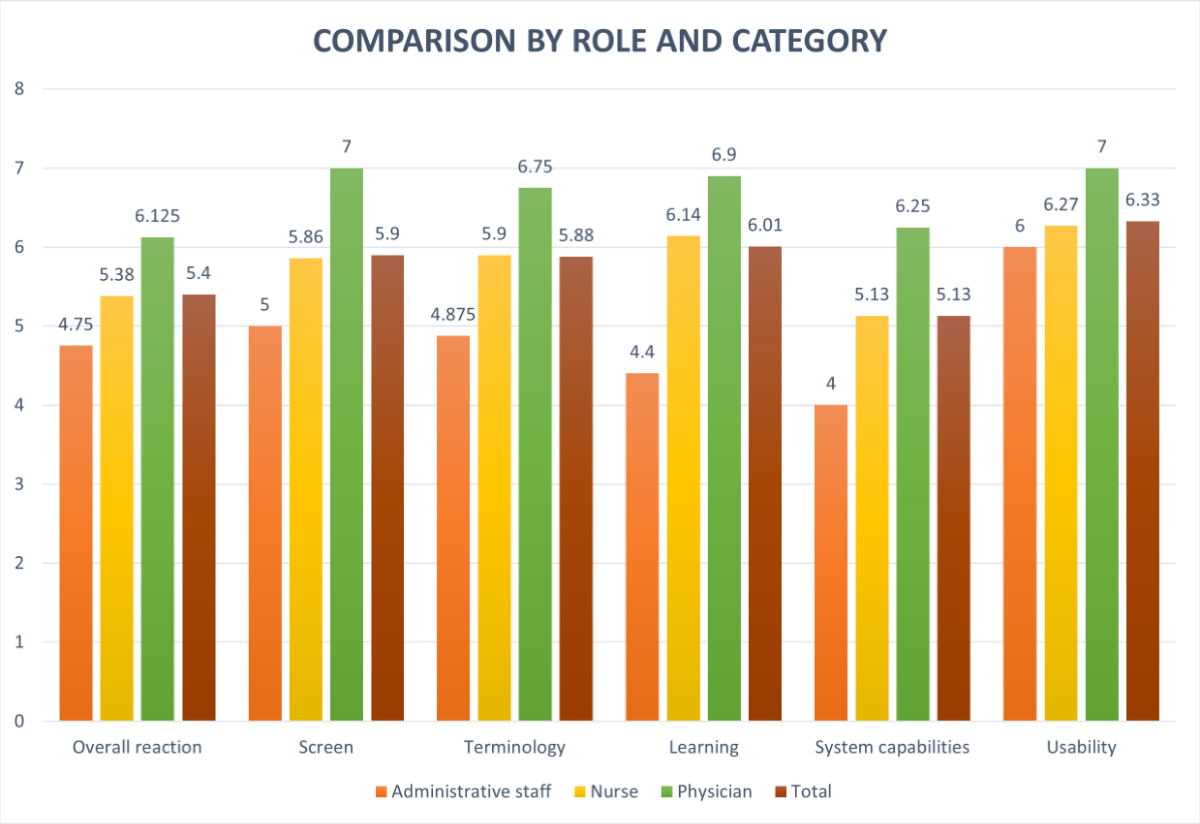
Comparison by patient subgroup and category of Questionnaire for User Interaction Satisfaction. For each question, the evaluation for each user group and the total study population is presented.

The means and SDs for each patient subgroup, and for the total population, were calculated for each item of the QUIS (Table 2). Also, for this questionnaire, the results for the nurse and physician groups were higher than those for the administrative staff, with mean scores of 5.64 (SD 1.7), 6.92 (SD 1.7), and 4.73 (SD 1.0), respectively. The median for the total group was 5.77 (IQR 4-7). Moreover, we calculated and graphically displayed the median for each category, providing an overall profile of areas that participants identified as a being particularly good or bad. As presented in Figure 12, usability (mean 6.33, SD 0) and learning (mean 6.01, SD 0.39) were the components with the highest scores considering the total population (among physicians, the highest scores were usability and screen design). The overall score represents a good level of satisfaction from users, with some aspects that should be improved.

**Table 2:** Questionnaire for User Interaction Satisfaction (QUIS) scores obtained for different user groups and the total study population.

QUIS item	Nurse, mean (SD)	Physician, mean (SD)	Administrative staff, mean (SD)	Total, mean (SD)
Overall reaction: terrible/wonderful	5.2 (1.6)	6.3 (1.1)	4 (0)	5.2 (1.5)
Overall reaction: difficult/easy	5.3 (1.7)	6.3 (2.0)	4.5 (0.7)	5.4 (1.6)
Overall reaction: dull/stimulating	5.4 (1.8)	7 (2.6)	5.5 (0.7)	5.7 (1.9)
Overall reaction: rigid/flexible	5 (1.6)	6 (2)	5 (0)	5.2 (1.5)
Screen readability	5.8 (1.3)	7.3 (2)	5.5 (2.1)	6.0 (1.6)
Highlighting usefulness	6.3 (1.7)	7 (2)	5 (1.4)	6.2 (1.7)
Information organization	5.4 (1.6)	7 (2)	5 (0)	5.6 (1.6)
Screen sequence clarity	5.4 (1.8)	7 (2)	4.5 (0.7)	5.6 (1.8)
Terminology consistency	5.8 (1.5)	7 (2)	4.5 (0.7)	5.8 (1.6)
Terminology relevance	6.3 (1.4)	7 (2.6)	5.5 (2.1)	6.3 (1.7)
Message positioning	5.5 (2.0)	7 (2.6)	5 (1.4)	5.7 (2.0)
Input prompt clarity	5.4 (2.0)	7 (2)	4.5 (0.7)	5.6 (1.9)
Ease of learning	6.6 (1.7)	7.3 (2)	4.5 (0.7)	6.4 (1.8)
Trial-and-error learning	5.3 81.4)	7 (2)	5 (0)	5.6 (1.5)
Command recall	6.2 (1.8)	7 (2)	5 (0)	6.2 (1.7)
Straightforward task performance	6.2 (1.5)	7.3 (2)	4.5 (0.7)	6.2 (1.6)
Reference material clarity	5.7 (1.7)	7 (2.6)	3 (1.4)	5.6 (2.0)
System speed	5.5 (2.1)	6.6 (2.3)	4.5 (0.7)	5.6 (2.0)
System reliability	4.3 (2.3)	6.6 (1.5)	3.5 (0.7)	4.6 (2.3)
Usability and UI	6.1 (1.9)	7.3 (2.0)	6 (1.4)	6.3 (1.8)
Total	5.64 (1.7)	6.92 (1.7)	4.73 (1.0)	5.77 (1.8)

### SAI Evaluation

**Figure 13:**
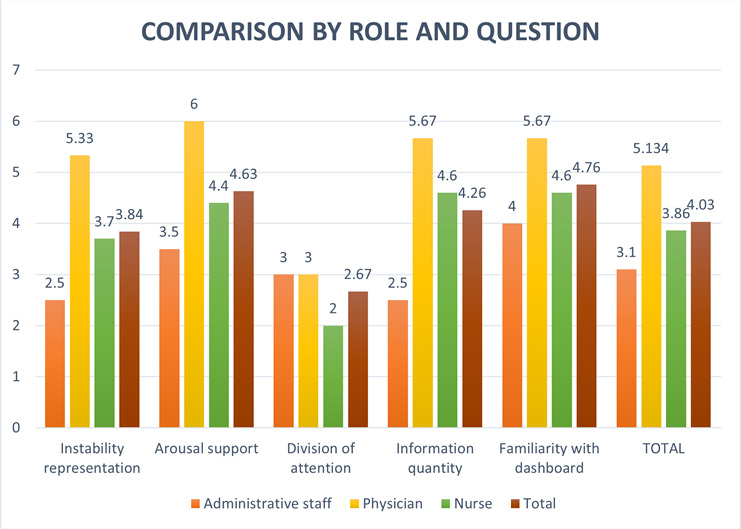
Comparison by patient subgroup and the question of Situation Awareness Index. For each question, the evaluation for each user group and the total study population is presented.

The mean overall SAI score was 4 (SD 1.36; Table 3). The SAI implies a person’s awareness regarding peculiar circumstances that arise through their interaction with the dashboard. This may have influenced the mean score for each subgroup; in fact, physicians obtained a total mean score of 5.13 (SD 0.9), much higher than that of the administrative staff (mean 3.1, SD 0.42), who used the dashboard less frequently. In general, the higher scores were obtained in “arousal support” and “familiarity of dashboard” items, with mean scores of 4.6 (SD 1.8) and 4.73 (SD 1.66), respectively (Figure 13). This result suggests relatively strong user awareness.

**Table 3:** Situation Awareness Index (SAI) scores obtained for different user groups and the total study population.

SAI item	Nurse, mean (SD)	Physician, mean (SD)	Administrative staff, mean (SD)	Total, mean (SD)
Instability representation	3.7 (2.11)	5.33 (0.58)	2.5 (0.71)	3.87 (1.92)
Arousal support	4.4 (1.84)	6 (1.73)	3.5 (0.71)	4.6 (1.80)
Division of attention	2 (1.76)	3 (2.64)	3 (0)	2.33 (1.83)
Information quantity	4.6 (2.01)	5.67 (1.53)	2.5 (1.41)	4.53 (1.95)
Familiarity with dashboard	4.6 (1.78)	5.67 (1.53)	4 (1.41)	4.73 (1.66)
Total	3.86 (1.43)	5.13 (0.90)	3.1 (0.42)	4 (1.36)

### Other Suggestions

Participants had the opportunity to add comments about possible dashboard improvements using a free-text box proposed at the end of the survey. Two participants filled out the box with the following comments:

E’ uno strumento indispensabile in una centrale come la nostra, è utile alla gestione del percorso. Mostra la prospettiva di dimissione contestuale durante tutto il ricovero, manca affinare l’aspetto qualitativo con l’utilizzo di indicatori specifici.”

Translation: It is a key tool for our unit and helps manage the patient pathway. It offers an overview of the planned discharge throughout the hospital stay, although the qualitative aspect could be improved by using specific indicators.

La non contestualità della presa in carico dovuta all’aggiornamento induce in errore nel mancato inserimento. Ogni volta che modifichi un dato, ritorna alla pagina iniziale e per verificare l’effettiva modifica devi cercare nuovamente il paziente.

Translation: “The lack of real-time updates in patient management can be confusing and may lead to missing data. Each time information is changed, the system returns to the main page, and the patient has to be searched again to check that the update was applied.”

The comments are focused both on the improvement of dashboard functionality, such as the page refresh after data entry, and on management aspects linked to patient presence in the dashboard after the first CCC consultation. In fact, nurses reported that they would prefer to see patients in the dashboard immediately after an order is sent by the ward and before visiting patients, in order to have more information available beforehand. Moreover, the first comment highlights the importance of the dashboard for monitoring and managing patients, as well as the possibility of inserting important patient information and having everything within a single view, such as the discharge setting.

## Discussion

### Overview

We developed a monitoring and management dashboard for the CCC group, and it remained in use after 1 year. Physicians and nurses evaluated the usability of the dashboard as “OK to good,” the satisfaction as acceptable, and good situation awareness. Clearly, the results and the suggestions from the users recommend continuous improvement of the dashboard, using a more efficient programming language and more accurate functionality. The obtained results, considering that this is the first dashboard release and that the clinicians were used to work with an Excel file, are satisfactory and, at the same time, allow for significant improvements in the dashboard functionality. First, R Shiny was used because it is an effective programming language for developing prototypes and allows rapid modifications; however, the next release will be developed using higher-performance languages, such as Angular or .NET. Moreover, all the user suggestions will be integrated into the next release, with the aim of implementing the requested improvements. We are already working to release the new version as soon as possible. Second, 3 months of use may have been too short a period to learn how to use the dashboard properly and get used to it. In fact, the change in workflow was challenging, and some clinicians found it difficult to adapt.

Finally, we are confident that after longer use and the integration of requests made by clinicians, the results in terms of usability and interaction, as well as awareness of the tool, will improve significantly.

At the end of the first dashboard release, we can consider ourselves satisfied with the results; in fact, beyond the questionnaire evaluation results, one of the most important objectives was the continuous use of the dashboard. Clinicians appreciated the proposed solution, which minimized the waste of time spent compiling data manually and the possibility of adding interactive and useful widgets.

### Principal Findings

Our study illustrates the development, implementation, and evaluation of an interactive dashboard for patient monitoring and management for the CCC group at Fondazione Policlinico Gemelli, Rome. First, we started from their actual workflow to understand which patient information was important and to identify which of these data could be automatically extracted from the DWH. We built a CCC Data Mart that was used as input for an extraction, transformation, and loading to correctly organize the data for the dashboard.

We proposed a first prototype of the dashboard after collecting the CCC manager’s requests, such as the layout, the functionality, and visual alerts. A total of 82% (23/28) of the data were automatically inserted into the dashboard, and the implementation of a pop-up allowed clinicians to insert the other ones. Moreover, the dashboard contained 3 panels to report important KPIs, useful for evaluating the number of patients in charge, and to facilitate the managerial aspects of the unit.

After 3 months of use, without the Excel file used previously, we proposed a survey to evaluate the dashboard’s usability, the users’ satisfaction, and their task efficiency. We proposed SUS, a highly validated usability test, and obtained a mean score of 61.5 (SD 15.7), considering all the participants. This result could imply “marginally high acceptability” with “OK to good” usability. This mean score changed considering the 3 subgroups, 63 (SD 18.2), 53.75 (SD 5.3), and 61.6 (SD 11.8) for nurses, administrative staff, and physicians, respectively. The obtained results show slightly lower satisfaction from the users compared with other studies in which clinical dashboards were developed [23-26]. In these studies, the proposed dashboards represented upgrades of previously developed prototypes. Therefore, the improved results may also have been influenced by previous interactions with users.

In this study, we also used QUIS to test user satisfaction, obtaining a total mean score of 5.77 (SD 1.8) for the entire user population, higher than the acceptance level of 5. Also in this case, nurses and physicians reached higher mean scores, 5.64 (SD 1.7) and 6.92 (SD 1.7), respectively, than the administrative staff. In general, the obtained evaluations show acceptable levels and are comparable with other studies in which clinical dashboards were evaluated [27-29].

Finally, we calculated the SAI to evaluate users’ task efficiency. The overall SAI score was 4, higher in the physician and nurse group than in the administrative staff. Compared with other studies that used SAI to evaluate the situation awareness [2,22], we obtained better results, demonstrating a high level of satisfaction among users.

### Limitations

First, the center already had a well-established procedure that, although associated with some errors due to manual input, allowed nurses to monitor patients. The transition to the dashboard obviously required a learning period to understand the functions and become accustomed to the new approach. Clinicians had to adapt to the dashboard, which allowed them to access most data in an automated way, but did not guarantee real-time updates, because updates occurred only twice daily. Moreover, the limited time available to test the dashboard may not have guaranteed complete familiarity with the system.

Another possible limitation of this study is the low number of participants who evaluated the dashboard. We proposed the comparison of results across different user subgroups, and this may appear limited because of the small sample size. In this case, we proposed a simple numeric comparison rather than a statistical analysis, for which larger groups are essential. Moreover, we cannot add other participants because the use of this dashboard is restricted to the CCC clinicians.

Regarding proposed questionnaires, SAI has not been validated, so its interpretive value is limited. However, the questionnaire was used previously in other studies to evaluate the situation awareness for dashboard users.

Ultimately, the most significant limitation is that this solution is the first integrated system used by physicians to manage patients. It is not always easy to change one’s workflow, especially when the number of patients is very high.

### Conclusions

In this study, we developed, implemented, and evaluated the CCC dashboard. The proposed questionnaires demonstrated acceptable performance, with possible improvements in both usability and design. The dashboard could also be implemented in other management departments within the hospital to monitor and manage different patient groups. The integration of this system could facilitate the work of various health care professionals by streamlining management processes and allowing them to focus more on patient care.

## Appendix

Multimedia Appendix 1 Italian version of dashboard mockup, dashboard screenshot and proposed questionnaires. Continuity of Care Centre data dictionary is proposed.

## Abbreviations

BRASS: Blaylock Risk Assessment Screening Score
CCC: Continuity of Care Centre
DWH: data warehouse
EHR: electronic health record
iCHECK-DH: Guidelines and Checklist for the Reporting on Digital Health Implementations
KPI: key performance indicator
QUIS: Questionnaire for User Interaction Satisfaction
SAI: Situation Awareness Index
SUS: System Usability Scale
