# Supplementary material for: An Interactive Digital Dashboard for Patient Monitoring and Management in a Continuity of Care Centre: Development and Preliminary Usability Evaluation Study
Source: JMIR Form Res. 2026 Jun 12;10:e81480. doi: 10.2196/81480 (PMC13309763; doi:10.2196/81480)
Supplement: Multimedia Appendix 1 [file formative_v10i1e81480_app1.docx]

**Multimedia Appendix 1**

This appendix provides the Italian version of dashboard muckup and the proposed questionnaires. Moreover, a comprehensive explanation of the CCC data dictionary with variables in Italian and English is proposed. Specifically, Figures from S1 to S9 offers an overview of the Italian version of dashboard mockup, in which each Figure is a specific page. Table S1, S2 and S3 show the Italian version of the proposed questionaries, specifically Table S1 shows SUS, Table S2 shows QUIS and Table S3 the Italian translation of SAI. Finally, Table S4 shows the CCC data dictionary, in which each dashboard’ variable is explain in Italian and English version, its format and the input type.

**
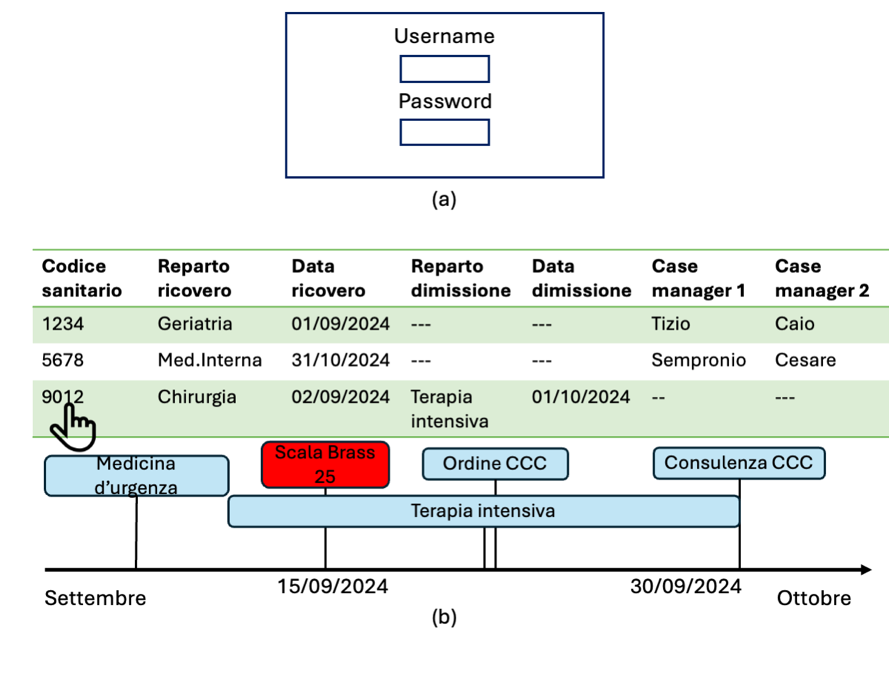
**

Figure S1. Italian version of mockup of the proposed dashboard prototype. (a) Login page, (b) First page.


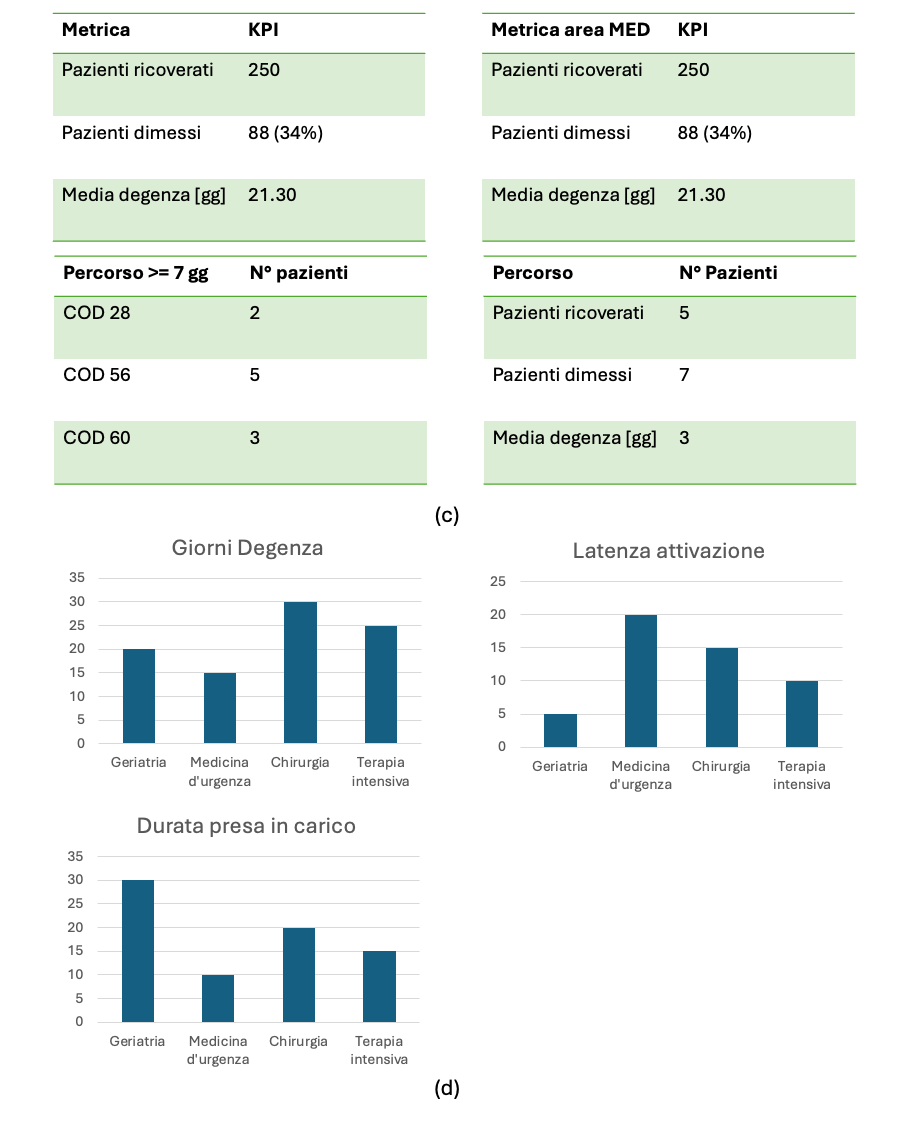


Figure S2. Italian version of mockup of the proposed dashboard prototype. (a) Second tab with KPIs tables. (b) Third tab with proposed graphs.


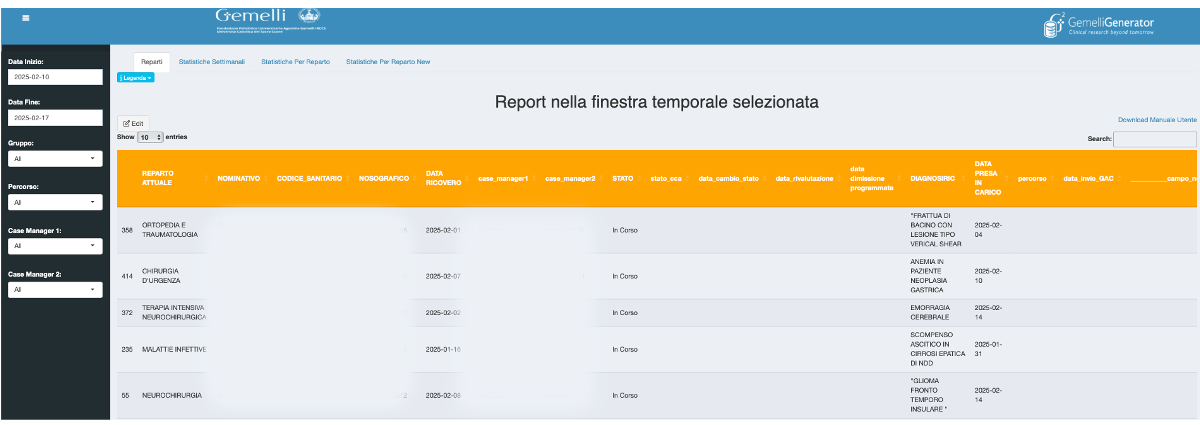


Figure S3. Italian version of first panel of the developed clinical dashboard. Each row represents a patient with several information, such as ID patient, actual ward, status, etc.


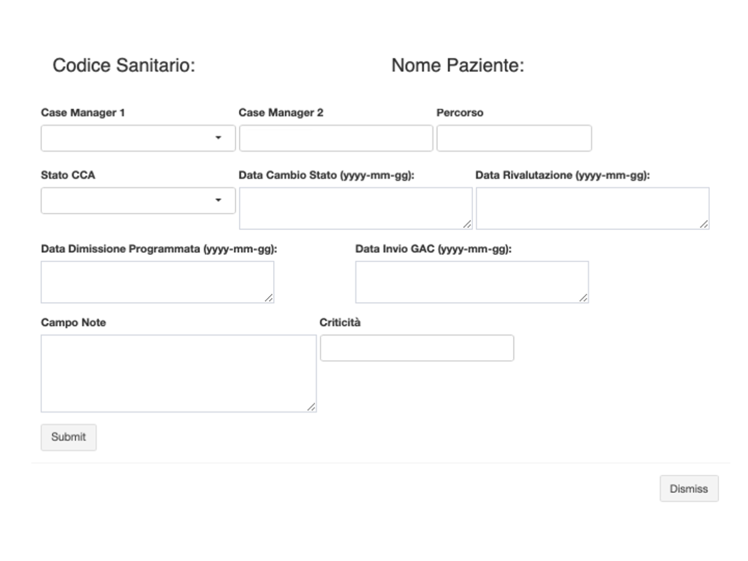


Figure S4. Italian version of pop up with insert form. Different information can be added via dropdown menu or free text.


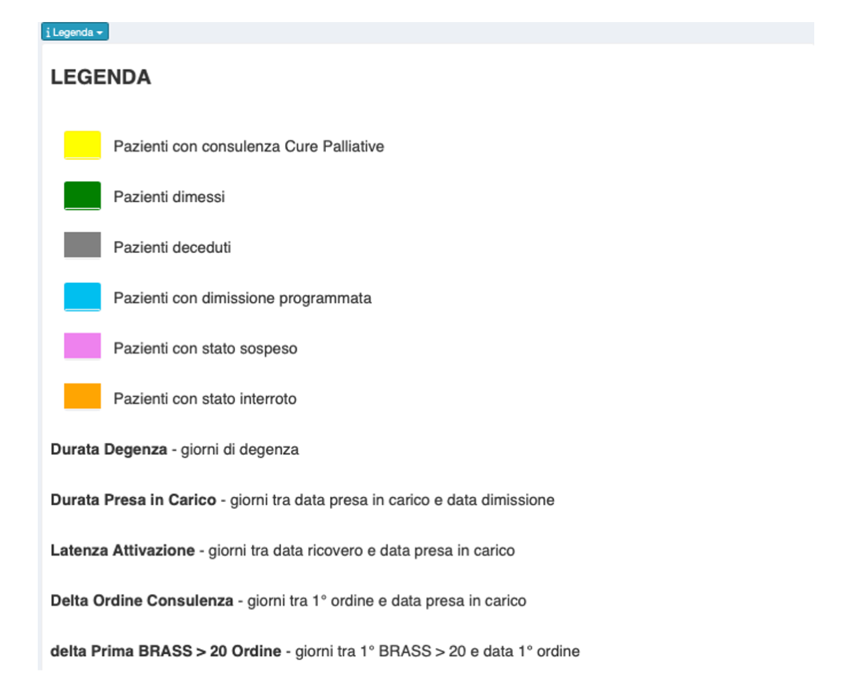


Figure S5. Italian version of legend with all possible visual alerts. Each color represents different patient’s critical issue.


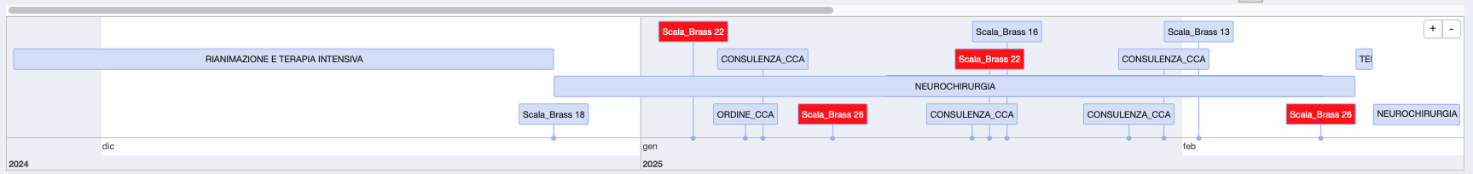


Figure S6. Italian version of patient’s timeline. Each CCC consultation, Brass scale, CCC order and transited ward are proposed in a compact view.


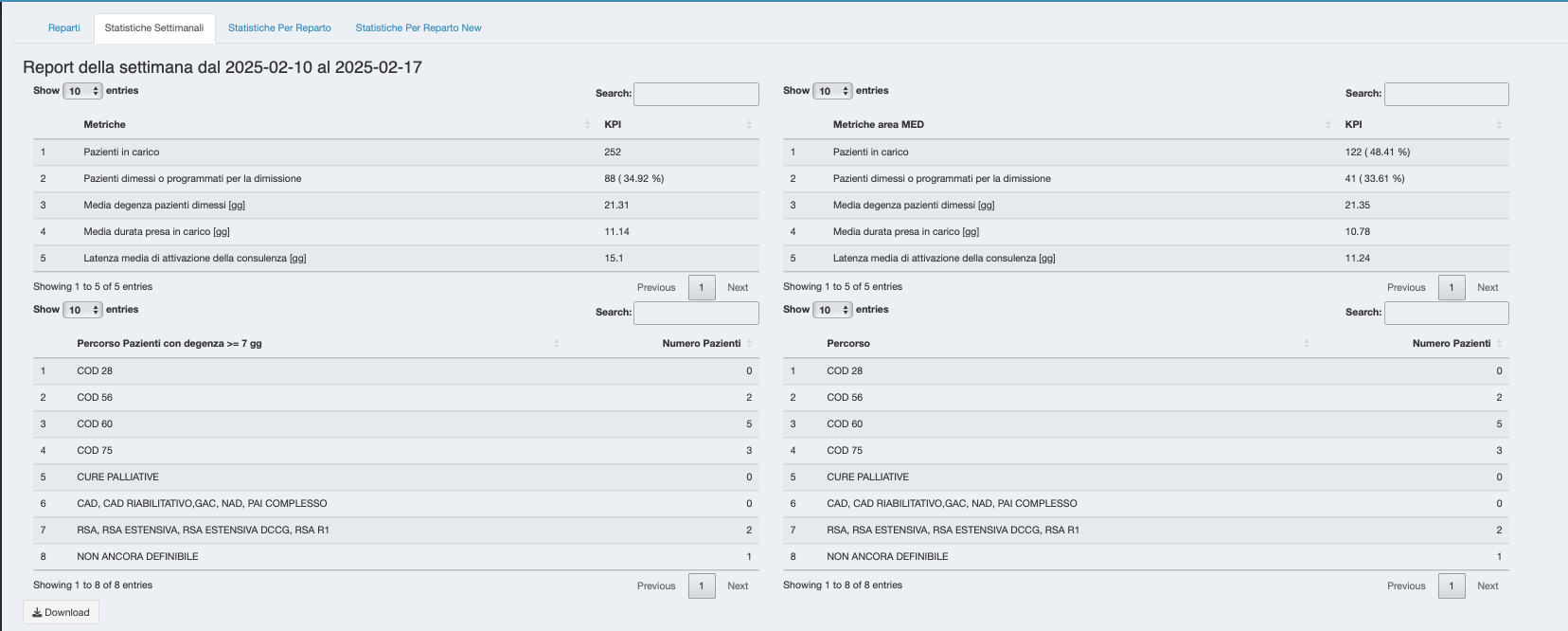


Figure S7. Italian version of second panel of clinical dashboard. Tables at the top represent general patients metrics; tables at the bottom represent number of patients for some the setting discharge.


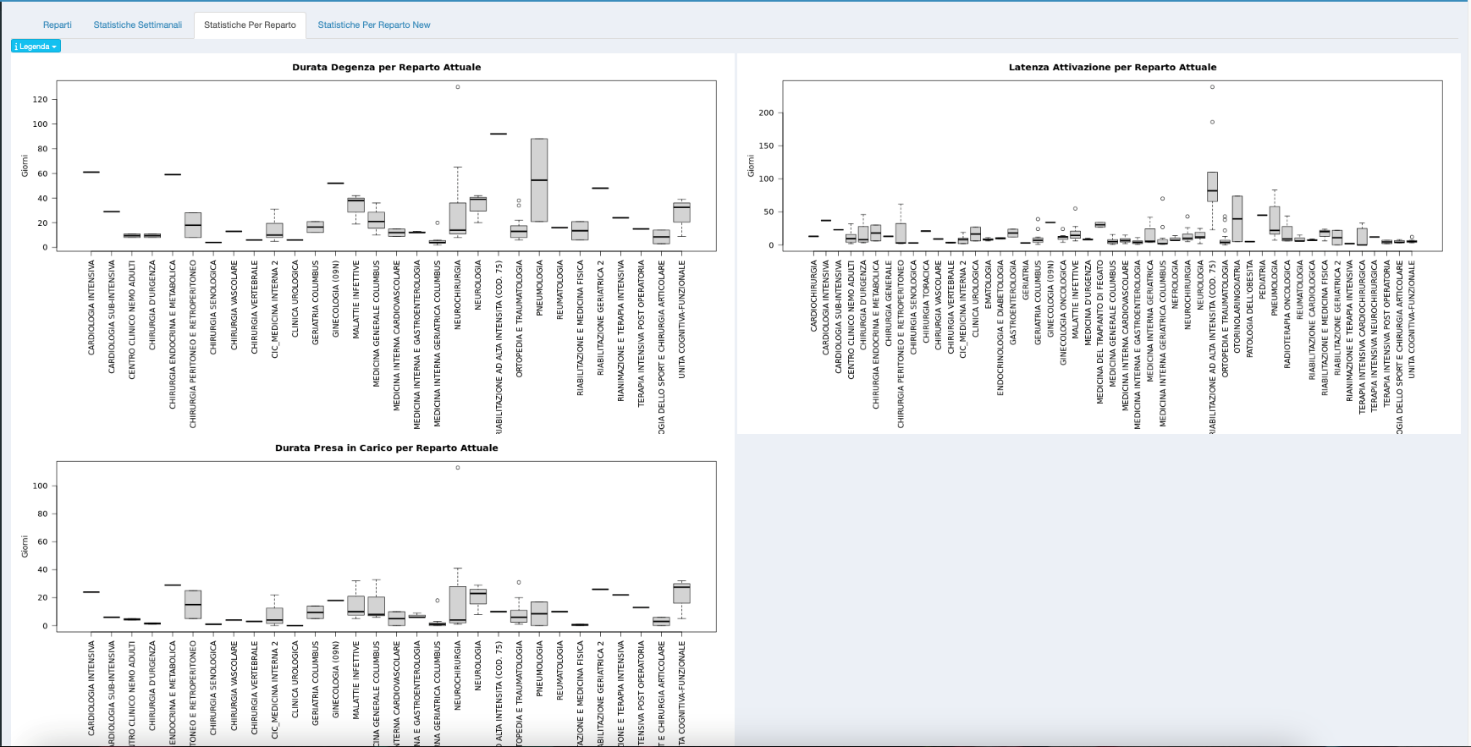


Figure S8. Italian version of third panel with three different graphs. Each graph represents a metric for different wards.


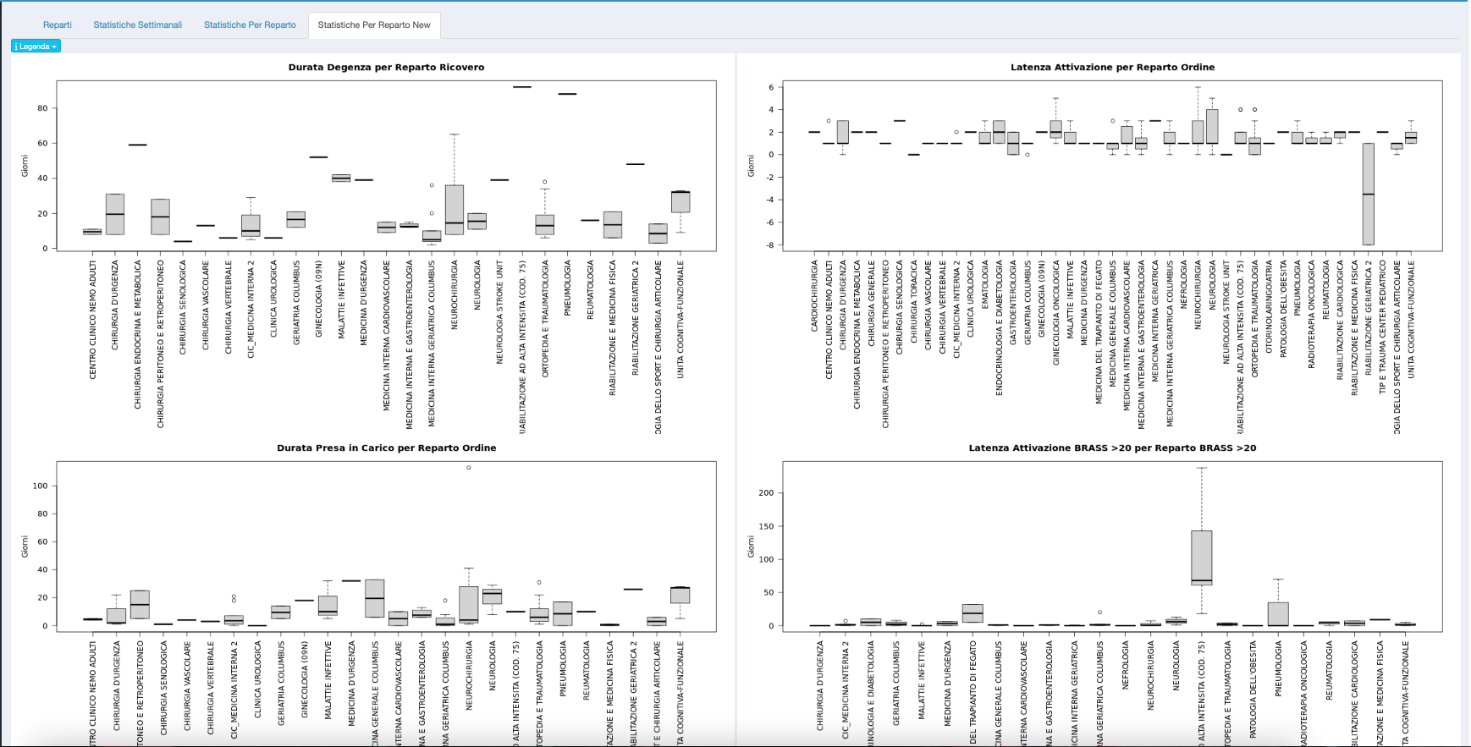


Figure S9. Italian version of fourth panel with four different graphs. Each graph represents a metric for different wards.

Table S1. Italian version of questionnaire proposed to the clinicians to assess the usability of the dashboard (12)

| **ID** | **Question** |
| --- | --- |
| 1 | Penso che mi piacerebbe utilizzare questo sistema frequentemente |
| 2 | Ho trovato il sistema complesso senza che ce ne fosse bisogno |
| 3 | Ho trovato il sistema molto semplice da usare |
| 4 | Penso che avrei bisogno del supporto di una persona già in grado di utilizzare il sistema |
| 5 | Ho trovato le varie funzionalità del sistema bene integrate |
| 6 | Ho trovato incoerenze tra le varie funzionalità del sistema |
| 7 | Penso che la maggior parte delle persone potrebbero imparare ad utilizzare il sistema facilmente |
| 8 | Ho trovato il sistema molto macchinoso da utilizzare |
| 9 | Ho avuto molta confidenza con il sistema durante l’uso |
| 10 | Ho avuto bisogno di imparare molti processi prima di riuscire ad utilizzare al meglio il sistema |

Table S2. Italian version of questionnaire proposed to the clinicians to assess the interaction satisfaction (15)

| **ID** | **Question** |
| --- | --- |
| 11  12  13  14 | ***Impressione generale del sistema***  Pessimo/Ottimo  Frustante/Soddisfacente  Monotono/Stimolante  Rigido/flessibile |
| 15  16  17  18 | ***Schermate***  I caratteri delle schermate del computer sono: difficili/facili da leggere  Il modo di far risaltare gli elementi sullo schermo è: inutile/utile  La disposizione delle informazioni sullo schermo è: illogica/logica  Sequenza delle schermate: confusa/chiara |
| 19  20  21  22 | ***Terminologia e informazioni sul sistema***  Uso della terminologia nel sistema: incoerent/coerente  La terminologia è appropriata alle attività che  stai svolgendo? mai/sempre  Posizione delle istruzioni sulla schermata: privo di logica/coerente  I messaggi di errore sono espressi in modo: sgradevole/gradevole |
| 23  24  25  26  27 | ***Apprendimento***  Apprendere ad utilizzare il sistema: difficile/facile  Esplorazione di funzioni per tentativi ed errori: difficile/facile  Ricordare il nome e l’uso dei comandi: difficile/facile  I compiti possono essere eseguiti senza  complicazioni: mai/sempre  Il materiale a supplementare di riferimento: confuso/chiaro |
| 28  29 | ***Capacità del sistema***  Velocità del sistema: troppo lento/ veloce  Il sistema è affidabile: mai/sempre |
| 30 | ***Materiale Multimediale***  I colori usati rispecchiano quelli reali: mai/sempre |

Table S3. Italian version of questionnaire proposed to the clinicians to assess the task efficiency (16).

| **ID** | **Construct** | **Question** |
| --- | --- | --- |
| 31  32  33  34  35 | Rappresentazione complessità  Stimolazione  Perdità di attenzione  Quantità informazioni fornite  Familiarità d’uso della dashboard | La dashboard rappresenta adeguatamente la complessità della CCC?  La dashboard mi aiuta a essere vigile e chiaro?  La dashboard distrae da alcune importanti attività della CCC?  La quantità di informazioni fornite dalla dashboard è appropriata per l'esecuzione delle attività della CCC?  Sono in grado di eseguire attività in modo più efficiente utilizzando la dashboard? |

Table S4. Data dictionary of CCC dashboard. Following acronyms explain the format and input type: char: character; num: numeric; DWH: DataWare-House.

| **Variable ITA** | **Variable ENG** | **Variable format** | **Input type** |
| --- | --- | --- | --- |
| NOSOGRAFICO | ID hospitalization patient | char | DWH |
| CODICE_SANITARIO | ID patient | char | DWH |
| REPARTO ATTUALE | Actual ward | char | DWH |
| DATA RICOVERO | Hospitalization date | date | DWH |
| STATO | Status | char | DWH |
| DATA PRESA IN CARICO | Take in charge date | date | DWH |
| DATA DIMISSIONE | Discharge date | date | DWH |
| REPARTO DIMISSIONE | Discharge ward | char | DWH |
| REPARTO RICOVERO | Hospitalization ward | char | DWH |
| DATA ORDINE | Order date | date | DWH |
| REPARTO ORDINE | Order ward | char | DWH |
| DATA PRIMA BRASS >20 | First Brass > 20 date | date | DWH |
| REPARTO BRASS >20 | Brass >20 ward | char | DWH |
| DURATA DEGENZA | Length of stay | num | DWH |
| DURATA PRESA IN CARICO | Length of taken in charge | num | DWH |
| LATENZA ATTIVAZIONE | Activation latency | num | DWH |
| DELTA ORDINE CONSULENZA | Delta consultation-order | num | DWH |
| DELTA PRIMA BRASS >20 ORDINE | Delta 1° Brass >20-order | num | DWH |
| case_manager1 | case manager1 | char | DWH |
| case_manager2 | case manager2 | char | DWH |
| percorso | Setting | char | Manually |
| criticita | Criticality | char | Manually |
| campo_note | Notes | char | Manually |
| data_cambio_stato | Change status date | date | Manually |
| stato_cca | CCC status | num | Manually |
| area | Medical area | char | DWH |
| NOMINATIVO_CODIFICATO | Patient name (encoded) | char | DWH |
| DIAGNOSIRIC | Hospitalization diagnosis | char | DWH |
